# Mid-infrared supercontinuum-based Fourier transform spectroscopy for plasma analysis

**DOI:** 10.1038/s41598-022-13787-w

**Published:** 2022-06-10

**Authors:** R. Krebbers, N. Liu, K. E. Jahromi, M. Nematollahi, O. Bang, G. Woyessa, C. R. Petersen, G. van Rooij, F. J. M. Harren, A. Khodabakhsh, S. M. Cristescu

**Affiliations:** 1 Life Science Trace Detection Laboratory, Department of Analytical Chemistry and Chemometrics, Institute for Molecules and Materials, Radboud University, 6525 AJ Nijmegen, the Netherlands; 2 Laser Spectroscopy and Sensing Laboratory, School of Physics and Materials Science, Anhui University, Hefei, 230601 China; 3 DTU Fotonik, Department of Photonics Engineering, Technical University of Denmark, 2800 Kgs. Lyngby, Denmark; 4 NORBLIS ApS, Virumgade 35D, 2830 Virum, Denmark; 5 NKT Photonics A/S, Blokken 84, 3460 Birkerød, Denmark; 6 DIFFER - Dutch Institute for Fundamental Energy Research, De Zaale 20, 5612AJ Eindhoven, The Netherlands; 7 Faculty of Science and Engineering, Maastricht University, Paul Henri Spaaklaan 1, 6229 GS Maastricht, The Netherlands

**Keywords:** Infrared spectroscopy, Plasma physics

## Abstract

Broadband mid-infrared (MIR) spectroscopy is a well-established and valuable diagnostic technique for reactive plasmas. Plasmas are complex systems and consist of numerous (reactive) types of molecules; it is challenging to measure and control reaction specificity with a good sensitivity. Here, we demonstrate the first use of a novel MIR supercontinuum (SC) source for quantitative plasma spectroscopy. The SC source has a wide spectral coverage of 1300–2700 cm^−1^ (wavelength range 3.7–7.7 μm), thus enabling broadband multispecies detection. The high spatial coherence of the MIR SC source provides long interaction path lengths, thereby increasing the sensitivity for molecular species. The combination of such a SC source with a custom-built FTIR spectrometer (0.1 cm^−1^ spectral resolution) allows detection of various gases with high spectral resolution. We demonstrate its potential in plasma applications by accurate identification and quantification of a variety of reaction products (e.g. nitrogen oxides and carbon oxides) under low-pressure conditions, including the molecular species with overlapping absorbance features (e.g. acetone, acetaldehyde, formaldehyde, etc.).

## Introduction

One of the most promising emerging methods for reducing carbon dioxide (CO_2_) emissions in chemical industrial processes is plasma-based gas conversion^1^. This is particularly of interest for the conversion of two common greenhouse gasses, CO_2_ and methane (CH_4_) into carbon monoxide (CO) and hydrogen (H_2_), also known as syngas^2,3^. Although traditional (catalytic) pyrolysis and dry reforming methods are considered efficient ways to produce syngas and oxygenates from CO_2_ and CH_4_, high temperatures and pressures are usually required^4–6^. To meet these requirements, the energy is largely supplied by burning fossil fuels, which is therefore accompanied by high CO_2_ emissions. Compared to thermal methods, discharges can be powered by fully-sustainable green energy, avoiding CO_2_ release^1,7–12^. However, the plasmas formed in electric discharges are complex systems, consisting of numerous (reactive) species, and it is a challenge to control its reaction specificity. Therefore, it is of great importance to develop a detection system with good sensitivity and specificity to analyze the reaction products from the discharge for different plasma conditions (e.g., different feed gases, electric discharge power and mixing ratios).

The preferred analytical method for characterizing reaction products of an electric discharge is absorption spectroscopy^13–15^, since it can combine high sensitivity and selectivity with excellent time resolution. The mid-infrared (MIR) wavelength region (2.5–25 µm) is of particular interest to the spectroscopy community, as many molecular gas species exhibit strong and unique absorption features in this region. Therefore, several different types of MIR sources are used for plasma diagnostics, such as MIR thermal sources (lamps)^15,16^, tunable diode lasers^14^, Quantum Cascade Lasers (QCLs)^17–19^, Interband Cascade Lasers (ICLs)^20^ and optical frequency combs^21,22^. While narrowband lasers, i.e., tunable diode lasers, ICLs, and QCLs, are well suited for sensitive detection of a specific molecule, they lack the ability to easily detect different types of molecules simultaneously.

Broadband absorption techniques, on the other hand, can detect and identify multiple species simultaneously, as is the case for the classical Fourier Transform InfraRed (FTIR) spectrometers, based on IR lamps^23^. However, the omnidirectionality and divergence of light from these sources limits the optical interaction path length with the gas sample species. Moreover, the low spectral brightness of these sources requires the spectrum to be averaged over longer periods to achieve an acceptable signal-to-noise ratio (SNR), especially for high spectral resolution measurements.

Optical frequency combs overcome these limitations but have (with some notable exceptions^24^) a relatively narrow spectral coverage. Furthermore, their high price and technical complexity limit their widespread use by end-users in real-life applications.

MIR supercontinuum (SC) sources are very suitable for broadband absorption spectroscopy, as they provide high spatial coherence, high spectral brightness, and ultra-broadband spectral coverage, outperforming conventional thermal sources and even some synchrotrons^25–28^. SC sources have demonstrated powerful capabilities in emitting in the visible and near-infrared range using silica fibers. Sources emitting up to 4 μm using fluoride fibers have become commercially available in recent years^29^. Several applications for these type of SC sources have been demonstrated, such as absorption spectroscopy with high sensitivity and selectivity for simultaneous measurement of multiple compounds^30^, spectroscopic standoff detection^31^, or optical coherence tomography in the MIR range providing real-time and high-resolution images^32,33^. Current developments in MIR SC sources extend the spectral range of the source beyond 4 μm to cover a larger part of the MIR fingerprint region^34^. Applications in absorption spectroscopy^35^ and hyperspectral imaging^36,37^ have been demonstrated for these highly experimental, new sources. Moreover, as the technology matures, this type of MIR SC sources is expected to be used for compact and cost-effective air quality sensor networks^38^.

SC sources in the visible and near-infrared wavelength region have been previously used for plasma diagnostics^39^, but to our knowledge, this report is the first demonstration of plasma diagnostics using a MIR SC source. Here, we demonstrate the advantages of MIR SC sources as broad spectral coverage (3.7–7.7 μm) and high sensitivity, achieved by the long optical path length of the spatially coherent beam.

## Results

### Experimental setup

**Figure 1:**
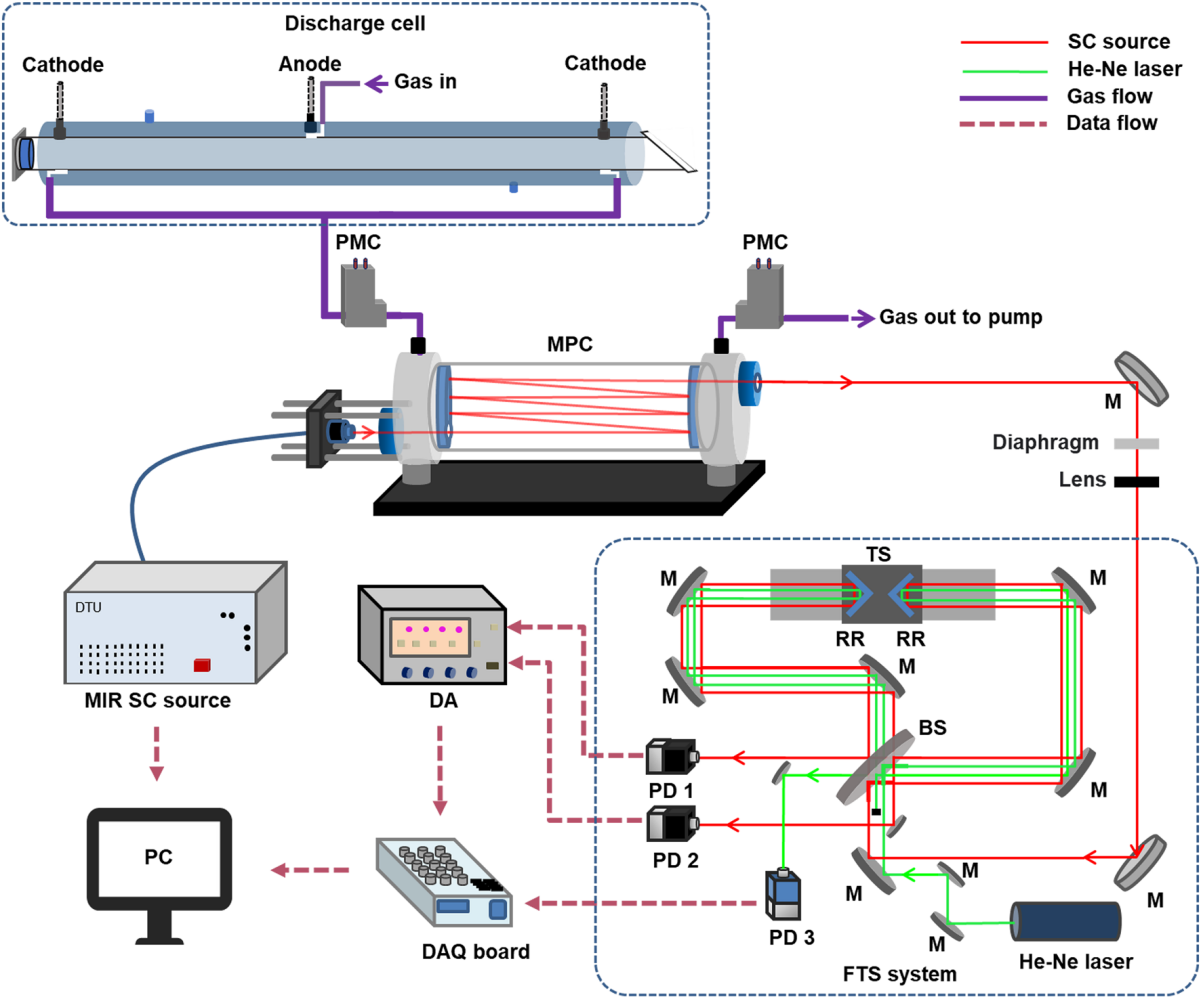
Experimental setup of the SC source and FTS-based multi-species detection system. *MPC* multipass cell, *PMC* pressure meter & controller, *M* mirror, *TS* translation stage, *RR* retroreflector mirror, *BS* beamsplitter, *He–Ne laser* helium–neon laser, *PD* photodetector, *DA* differential amplifier, *DAQ board* data acquisition board, *PC* computer.

The developed spectroscopic system consists of the MIR SC source, whose beam transmits through a multipass cell (MPC) containing the products of the plasma reaction at a low pressure. The transmitted beam is sent towards a custom-built Fourier Transform Spectrometer (FTS) with a spectral resolution of 0.1 cm^−1^ (3 GHz), allowing detection of narrow molecular absorption lines of gas species at a low pressure. Outside the MPC, the SC beam path was shielded and purged continuously with N_2_ gas. The experimental setup is presented in Fig. 1. The light source is a newly developed, fiber-coupled MIR SC source (DTU Fotonik, average power ~ 86 mW, pulse duration ~ 0.5 ns, repetition frequency 3 MHz^40^). The MPC has a 31.2 m effective interaction length (Thorlabs, HC30L/M-M02) and is connected to the discharge cell, such that the reaction products of the plasma are sent to the MPC for interaction with the SC beam. The custom-built FTS has been demonstrated and discussed in detail in our previous work^35^. A brief description is presented in the Methods section, as well.

### Source characterization

**Figure 2:**
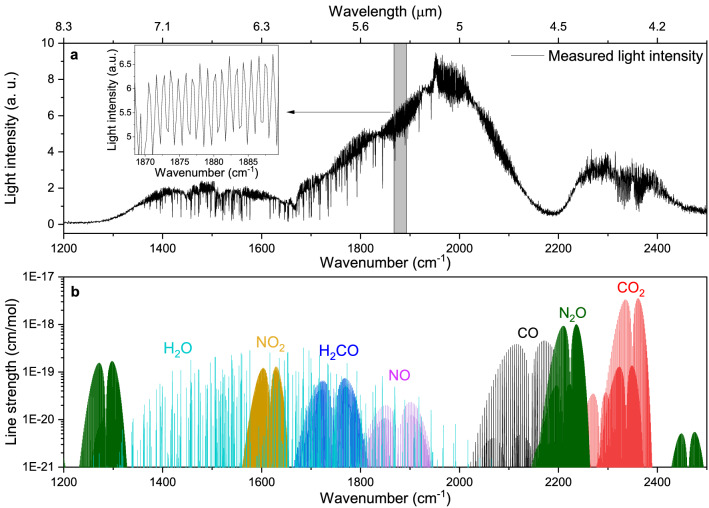
Spectral coverage of the MIR SC source and absorbing molecular species. (**a**) The spectrum of the MIR SC source with a spectral resolution of ~ 0.27 cm^−1^ (8 GHz) measured under vacuum conditions, insert: an enlargement of the spectrum, showing the etalon fringes in the measured spectrum. (**b**) Simulated spectral line intensity of different species from HITRAN2020 between 1300 cm^−1^ and 2500 cm^−1^.

The spectral coverage of the MIR SC source was characterized with the FTS system by sending the SC light through the MPC under vacuum (< 10^–3^ bar). The resulting power spectral density is shown in Fig. 2a and covers the spectral region between 1300 and 2700 cm^−1^ (3.7–7.7 μm). While most of the spectral power is between 1700 and 2100 cm^−1^, spectroscopy in the other wavelength ranges is still feasible with high SNR, due to the high average power of the MIR SC source and sensitive photodetectors. Highly absorbing water (H_2_O) and CO_2_ lines can be observed in the spectrum, despite the measurement being performed under vacuum conditions. In Fig. 2a, the etalon fringes are also visible in the spectrum due to the partial overlap of the SC spots of the consecutive reflections on the MPC mirrors. However, the measured absorbance spectrum is not affected as the etalon fringes are stable and are canceled out by normalizing the sample spectra by the background spectra^35^. The simulated spectral line intensities of the relevant species from the HITRAN2020 database^41^ between 1300 and 2500 cm^−1^ are shown in Fig. 2b. Due to the overlap of the absorption lines, a low pressure (16.5 mbar) and a high spectral resolution (3 GHz/0.1 cm^−1^) are required to distinguish between the absorption lines.

### System validation

**Figure 3:**
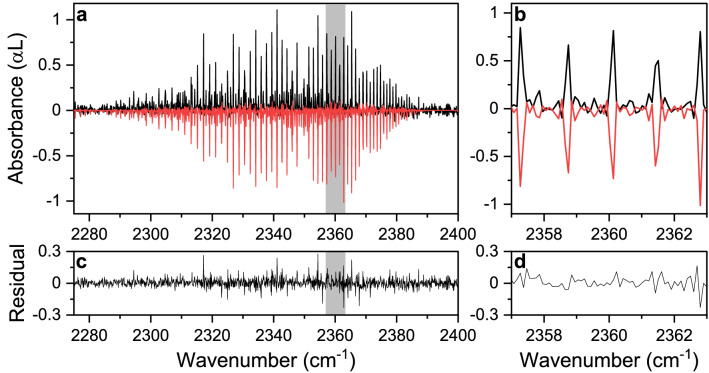
Experimental spectrum of CO_2_. (**a**) Measured spectrum (in black, 0.1 cm^−1^ spectral resolution, pressure 16.5 mbar, 500 averages in ~ 16 min) of 495 ppm CO_2_ diluted in N_2_ and a fitted modelled spectrum (in red, inverted) from the HITRAN database using a Voigt profile and convolved with a sinc-function. (**b**) Details of a part of the spectrum indicated in (**a**) as a grey rectangle. (**c**,**d**) Residual of the fit.

To initially evaluate the performance of the MIR SC FTS-based system, we measured the spectrum of a gas mixture of 495 ppm CO_2_ in N_2_ at 16.5 mbar pressure. Here, we dilute a calibrated mixture of 5% CO_2_ in N_2_ (Linde Gas) with pure N_2_, down to 495 ppm, using two flow controllers. The measured spectrum is shown in Fig. 3 (in black, 500 averaged scans in ~ 16 min, 1.9 s per scan) alongside a fitted, modelled CO_2_ spectrum (in red, inverted for clarity). The model spectrum is calculated using the HITRAN database parameters and a Voigt profile, convolved with a sinc function. The retrieved concentration from the fit is 485 ± 12 ppm. The uncertainty is calculated from the standard deviation of the noise in the residual of the fit. In Fig. 3a, the full rotational-vibrational band of CO_2_ is shown. To demonstrate the agreement between the measurement and the fitting routine, an enlargement of the spectral features between 2357 cm^−1^ and 2363 cm^−1^ is displayed in Fig. 3b. The residuals are shown in the bottom panels (Fig. 3c,d). The rather featureless residuals demonstrate the high precision of the frequency calibration, as well as the good quality of the fitting routine. The small peaks which are still visible in the residual can be contributed to the influence of carbon dioxide in the beam path outside the MPC. The light intensity at these specific peaks is reduced in both the background and measurement spectrum to an extent that it degrades the fitting quality.

**Figure 4:**
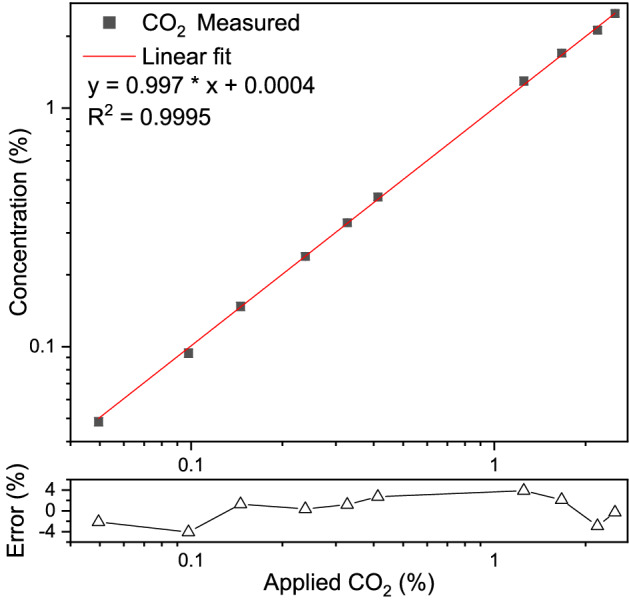
Retrieved concentrations of CO_2_ for various dilutions of CO_2_ in N_2_, along with a linear fit to demonstrate the linear response of the system. The error provided is the relative difference between the applied and retrieved concentration.

The linear response of the system to different applied CO_2_ concentrations was evaluated in a dynamic range between 0.05% and 2.5% of CO_2_ in N_2_, by diluting the 5% CO_2_ in N_2_ mixture further using pure N_2_ gas. Each measurement consists of 500 averaged spectra, measured in ~ 16 min. The reference spectra for the linear fitting routine were simulated from the HITRAN database as described before. The retrieved concentrations from the fit versus the applied concentrations are shown in Fig. 4, together with the corresponding errors, exhibited in the lower panel. The linear fit shows a Pearson correlation coefficient square value of 0.9995, demonstrating a very good agreement between the measured concentration values and the applied concentrations. The corresponding relative errors are within a ± 4% margin for the entire range, with an average error of 2%.

### Product analysis of CO_2_/N_2_ plasmas

**Figure 5:**
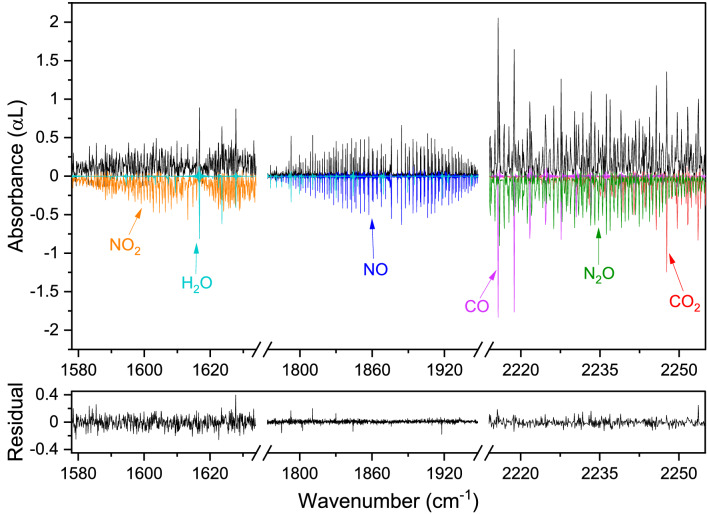
Measured spectrum (in black, 0.1 cm^−1^ spectral resolution, 16.5 mbar pressure, ~ 16 min) of reaction products of a 50% CO_2_/50% N_2_ discharge and the fitted modelled spectra (in colors, inverted) based on the HITRAN database parameters and Voigt profiles convolved with a sinc function. The residual of the fit is shown in the lower panel.

To further assess the performance of the system and to demonstrate its ability to quantify complex mixtures of reaction products, the outflow of a discharge was used. The discharge was generated in a flowing mixture of 50% CO_2_ in N_2_ (flow of 2 l_n_/h) in a discharge cell at a pressure of 25 mbar. The applied voltage was 17.5 kV with a current of 10 mA that results in a specific energy input of 7.1 MJ/mol. The outflow of the discharge cell was guided to the MPC, which had a controlled pressure of 16.5 mbar. In Fig. 5, the measured absorbance spectra of nitrogen dioxide (NO_2_), nitric oxide (NO), nitrous oxide (N_2_O), CO, CO_2_ and H_2_O are shown (in black) together with corresponding simulated spectra (inverted, in color, using HITRAN parameters). The concentrations of the species retrieved from fitting the simulations to the absorption spectra are shown in Table 1. The practically featureless residual of the fit, shown in the bottom panel of Fig. 5, indicates a very good fit for all detected species. The few spikes which remain visible in the residual can be attributed to the effect of highly absorbing water vapor lines in the atmospheric air outside the MPC, degrading the fitting quality.

**Table 1:** Retrieved concentrations of electric discharge products.

	Retrieved concentration (% or ppm)	
	50% CO_2_/50% N_2_	30% CO_2_/70% CH_4_
Carbon dioxide (CO_2_)	13.0 ± 0.6%	13.9 ± 0.7%
Carbon monoxide (CO)	14.6 ± 0.7%	32.1 ± 1.7%
Nitrous oxide (N_2_O)	570 ± 30 ppm	–
Nitrogen dioxide (NO_2_)	154 ± 13 ppm	–
Nitric oxide (NO)	0.33 ± 0.01%	–
Ethylene (C_2_H_4_)	–	0.73 ± 0.03%
Formaldehyde (H_2_CO)	–	0.13 ± 0.01%
Acetone (C_3_H_6_O)	–	0.3 ± 0.1%
Acetaldehyde (C_2_H_4_O)	–	0.3 ± 0.1%

A wide variety of different species are detected over a range of concentration levels from hundreds of ppm to percentage-level. The broad spectral coverage does not only allow for detection of absorption lines of various compounds, but also to select absorption lines of a certain species with an appropriate line strength for the given concentration, preventing limitations arising from absorption lines which absorb almost 100% of the light.

### Product analysis of CO_2_/CH_4_ plasmas

**Figure 6:**
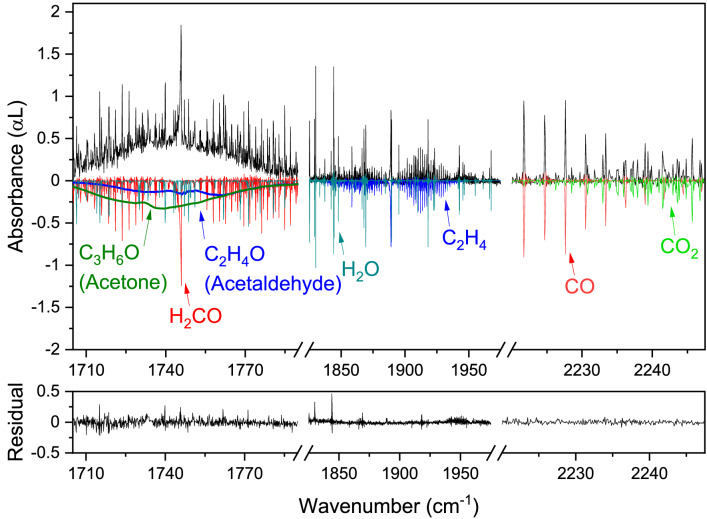
Measured spectrum of reaction products of a discharge of 70% CH_4_ and 30% CO_2_ (in black, 0.1 cm^−1^ spectral resolution, 16.5 mbar pressure, ~ 16 min). Reference spectra of formaldehyde, water, carbon monoxide, carbon dioxide (from HITRAN), acetone and acetaldehyde (from PNNL) and ethylene (from GEISA) shown inverted.

A bigger challenge for the spectroscopic analysis of a complex mixture of reaction products is the dry reforming of methane, as more complex molecules are formed, which cannot be found in HITRAN. Therefore, to extend the evaluation of the system, a CO_2_/CH_4_ discharge is generated. A discharge voltage of 18 kV with a current of 15 mA provided a specific energy input of 11 MJ/mol for a mixture of 70% CH_4_ and 30% CO_2_ (flow 2 l_n_/h, 19 mbar pressure). The absorbance features of the detected products are presented in Fig. 6. Here, a broad absorbance feature is visible in the 1700–1800 cm^−1^ wavenumber region. In general, this indicates the presence of molecular species with a large number of closely spaced rotational transitions in the vibrational band, which cannot be resolved spectroscopically at this pressure and temperature. Using the PNNL database, this specific absorbance profile was found to be likely belonging to acetone (C_3_H_6_O) and acetaldehyde (C_2_H_4_O), both with a concentration of 0.3 ± 0.1%. The concentration could however not be determined exactly, as PNNL is constructed with experimental data at 1 atmosphere pressure, which is significantly different from our experiment. We confirmed the presence of these two molecular species in our mixture using proton-transfer-reaction mass spectrometry (PTR-MS)^42^ and gas chromatography–mass spectrometry (GC–MS)^43^. Moreover, the residual shows the ability of the system to detect overlapping absorbance features of multiple species, as both the acetone, acetaldehyde, formaldehyde (H_2_CO) and most H_2_O spectral features are fitted well with their reference spectra. Furthermore, the absorbance lines of ethylene (C_2_H_4_) around 1880 cm^−1^ are not included in the HITRAN database. Therefore, the GEISA database^44^ was used to create a simulated reference spectrum for C_2_H_4_, indicating a concentration of 0.73 ± 0.03%.

In summary, this experiment demonstrates the system’s ability to accurately detect numerous molecular species created in electric discharges of CO_2_/CH_4_, even for the ones with overlapping spectral features.

### Plasmas with varying ratio of CO_2_ and CH_4_

**Figure 7:**
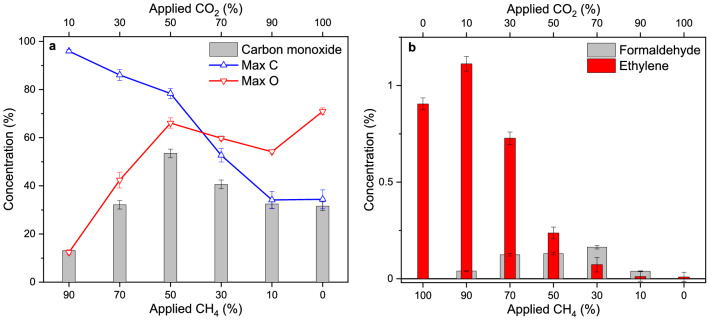
Retrieved concentrations of reaction products. (**a**) Measured CO concentrations for varying CH_4_/CO_2_ ratio generated by the plasma (in grey). Maximum amount of carbon atoms (Max C, blue) and oxygen atoms (Max O, red), available from the CO_2_ and CH_4_ depletion in the discharge, indicate the maximum CO concentration that can be generated. The measured CO-levels are below these values. (**b**) Measured concentrations of ethylene (in red) and formaldehyde (in grey) generated by the plasma for varying ratios of CH_4_/CO_2_.

To demonstrate the possibilities of the system for plasma analysis and study, a quantitative analysis of the products formed in the electric discharge is made, using a series of measurements with a varying CO_2_/CH_4_ ratio (gas flow 2 l_n_/h, 18 kV, 15 mA, specific energy input of 11 MJ/mol). In Fig. 7a, the measured CO concentration is displayed for varying mixtures of CO_2_/CH_4_, along with the assumed limit in CO production, calculated from the number of carbon (in red) and oxygen atoms (in blue) available in the system to react to CO. These limits are calculated from the retrieved CO_2_ concentrations, which are shown in Supplementary Fig. 1. From the difference between the CO_2_ concentrations with the discharge on or off, the maximum number of carbon and oxygen atoms available for conversion is determined. The highest CO production is found for a 50/50-mixture of CO_2_/CH_4_. The CO values are within the expected maximum available carbon and oxygen atoms generated in the plasma, indicating a correct reaction balance. As the concentration of CO (~ 53%) is higher than the concentration of converted CO_2_, this indicates that CO is not only formed by removal of an oxygen atom from CO_2_, but also by recombination of the removed oxygen atom from CO_2_ with a dehydrogenated carbon atom from CH_4_. Furthermore, there is additional production of C_2_H_4_ and H_2_CO (Fig. 6), of which the retrieved concentrations are presented in Fig. 7b.

## Discussion

A MIR SC FTS system is presented for the first time for plasma analysis. We showed that various molecular species of interest can be detected and quantified across the spectral range of the supercontinuum source, while the high spectral resolution from the FTS allowed for identification of molecular species with overlapping absorption features. Therefore, the MIR SC FTS system provides a unique set of characteristics that are especially useful for plasma diagnosis, combining the broad spectral coverage, high spectral power, and high spectral resolution with a high spatial coherence, stability and ease of use. The high detection sensitivity makes it possible to monitor the molecular reaction products in a low-pressure plasma, minimizing spectral interference between different species and accurately determining their concentrations.

A rather detailed comparison of the MIR SC FTS with other spectroscopy systems in the MIR range is presented in our previous work^35^. The noise equivalent absorption sensitivity of the MIR SC FTS is one to two orders of magnitude lower than MIR optical frequency comb-based spectrometers and it also provides coarser spectral resolution compared to comb-based systems. However, the broader spectral coverage of the MIR SC FTS allows simultaneous detection of a large number of molecular species. In addition, the ease-of-use, compactness, robustness, and lower cost of MIR SC sources make them better suited for real-life plasma applications.

Classical FTIRs can match the spectral coverage of our system. As mentioned elsewhere^35^, they currently provide a better Noise Equivalent Absorption Sensitivity compared to the MIR SC FTS presented here. However, classical FTIRs tend to operate at a lower spectral resolution, as the combination of high resolution with the relatively low spectral power density of a thermal source would increase the need for spectral averaging and consequently lengthen the measurement time. Most importantly, the thermal sources lack spatial coherence, making it challenging to create long interaction path lengths (e.g. by using an MPC with a long effective path length) to maximize the sensitivity. Furthermore, a well-collimated beam with a small beam diameter is required for in-situ monitoring in long (> 1 m) plasma cells, which is also difficult to obtain using thermal sources.

Our system’s performance can also be compared specifically to previous studies on plasma analysis using QCLs^20^ and tunable diode lasers^14^, which are currently the most frequently used laser-based systems for absorption spectroscopy on plasmas. Their narrow spectral range, which generally is in the order of a few wavenumbers (with some exceptions for external cavity QCLs), limits the number of detectable molecular species as well as the detection and identification of unknown compounds and introduces difficulties for overlapping/broadband spectra. For instance, detection, identification, and quantification of the overlapping spectra of acetaldehyde, acetone, and formaldehyde as in Fig. 6 would have been significantly more difficult with such a system.

In conclusion, the reaction products were quantified for a plasma with a varying ratio of CO_2_ and CH_4_. The CO yield was found to increase when CH_4_ was added to the CO_2_ plasma, which could be explained by the recombination of the removed oxygen atom from CO_2_ with a dehydrogenated carbon atom from CH_4_. The observed trends in the generation of the other observed products, H_2_CO and C_2_H_4_, demonstrated a good agreement with previous studies. H_2_CO should be composed from H-atoms of CH_4_ and O-atoms of CO_2_ and is indeed only found in mixtures containing both CH_4_ and CO_2_^45^. The formation of C_2_H_4_ increases towards higher concentrations of CH_4_ in the mixture and can be easily explained as C_2_H_4_ is a product from the dehydrogenation of CH_4_^46^.

Currently, the relative intensity noise of the MIR SC source limits the sensitivity of the spectrometer, although this is not essential for the demonstrated application, in identification and quantification of the products of a plasma reaction. Moreover, with the current rapid developments in MIR SC sources, it is anticipated that the intensity noise of these sources will be reduced further in the near future^29,34^. Finally, the spatial coherence of the MIR SC beam could also be exploited for other applications, such as for accurate in-situ probing in plasmas, to monitor the plasma dynamics, as well as to detect intermediate products in the plasma.

Finally, besides the application of this system for electrolytic chemical conversions, it can potentially be applied as a spectroscopic tool for many more plasma applications. For example, within the synthesis of carbon nanotubes using plasma-enhanced chemical vapor deposition (PECVD), monitoring a correct flow of reactants and the formation of gas phase intermediates and products is currently done with mass spectrometry^47^. Our system could replace mass spectrometry, providing on-line measurements and being able to distinguish species with identical masses. Furthermore, in plasma medicine cold atmospheric plasma jets are used to treat human skin or wounds^48^. Optical Emission Spectroscopy (OES) and FTIR are used to investigate the species formed in these plasmas. As OES lacks quantification^49^ and FTIR lacks sensitivity due to limited interaction lengths and spectral resolution^50^, a MIR SC FTS system might be preferred to quantify the species formed in plasma jets for these applications. Similarly, the system could be of interest for other plasma-based techniques, such as wastewater treatment^51^, or surface and material modification and sterilization^52,53^.

## Methods

### Fourier transform spectrometer

The overview of the full experimental system is presented in Fig. 1. The output fiber of the MIR SC source is connected to a reflective collimator that is mounted on the MPC to limit the optical path outside the MPC. After passing through the MPC, the MIR SC beam is aligned to the Fourier transform spectrometer (FTS) using two flat mirrors. A diaphragm is used to filter stray light and a long-focal-point lens was used to match the beam waist to the center of the translation stage in the FTS. The setup of the FTS system has been described in detail in our previous work^35^. Briefly, the FTS system is based on a Michelson interferometer; the incoming light beam is split by a beamsplitter into two arms, each leading to a hollow retroreflector mirror. The retroreflector mirrors reflect the beams with slight horizontal and vertical shifts, so that the reflected beams recombine on the beamsplitter and the two resulting superpositions of the beams (interfering pair of beams) can both propagate towards separate photovoltaic detectors (PVI-4TE-10.6, Vigo Systems). The two interference patterns at the output of the detectors are subtracted from each other by a differential amplifier (SR560, Stanford Research system) in a balanced detection scheme. Since the two interference patterns are out-of-phase, but the intensity noise is in-phase, the balanced detection scheme highly reduces intensity noise, which leads to an effective improvement of the signal-to-noise ratio (SNR). The two retroreflectors are mounted back-to-back on a linear-motor translator stage. Scanning the translator stage over a distance of 2.5 cm results in an optical path difference of 10 cm between the two beams, which provides a spectral resolution of 0.1 cm^−1^ (3 GHz). A He–Ne laser beam is sent along the SC beam path in the FTS to create an interferogram for a known and stable wavelength. The interferogram of the He–Ne laser is recorded by a separate photovoltaic detector. To prevent the interference of absorption by H_2_O and CO_2_ in the ambient air with the measurement, the FTS is confined in a closed box and continuously purged with N_2_ gas.

### Data analysis

For real-time analysis of the detected interferograms, a LabVIEW-based data processing system is developed. A flowchart of the algorithms used for this analysis is shown in Supplementary Fig. 2. The wavenumber calibration of the MIR SC source is performed through a resampling process using a He–Ne laser. In this process, explained in detail in Ref.^35^, the algorithm finds the zero-crossings of the He–Ne laser interferogram (whose beam is propagating in parallel to the MIR SC beam) by linear interpolation. Then, it linearly interpolates the MIR SC interferogram at the zero-crossing positions of the He–Ne laser interferogram. Therefore, the interpolated MIR SC interferogram in the zero-crossing domain is calibrated with an optical path difference step size equal to the zero-crossing intervals. After the calibration, a Fast Fourier Transform (FFT) of the resampled interferograms yields the spectra. Note that we used a natural boxcar apodization function for the interferograms. The absorbance spectrum is obtained by dividing the transmission spectrum of the sample to the background (the latter is taken after purging the MPC with pure N_2_ gas) and taking the natural logarithm. The absorbance spectra of the different species are simulated, using parameters from the HITRAN2020^41^ or GEISA^44^ database and convoluted with a sinc instrument lineshape function, to fit the natural boxcar apodization of the interferograms. GEISA was used to simulate the absorbance of ethylene (C_2_H_4_) around 1900 cm^−1^, since this ethylene band is not completely included in HITRAN. The PNNL^54^ database was used to identify absorbance from acetone (C_3_H_6_O) and acetaldehyde (C_2_H_4_O). However, PNNL contains actual FTIR measurements of absorbance spectra at atmospheric pressure, which cannot correctly be recalculated for low pressures. Finally, the concentrations of different species are calculated using a multiple linear regression algorithm based on the least squares method^35^. Given the limitations of the PNNL data, the concentration of acetone and acetaldehyde can only be approximated.

### Plasma conditions

For the generation of the plasma, a water-cooled discharge tube (length 180 cm, internal diameter of 1.5 cm) is used to create a uniform electric discharge. The anode and gas inlet are located in the center, while two hollow cathodes (4 cm long each) and the gas outlets are located at either end of the discharge tube. A current-stabilized high-voltage (HV) power supply (Haefely Hipotronics, US, providing up to 25 kV, 40 mA) is used for generating a direct current (DC) electric discharge in the tube^22^. The gas inflow is regulated with flow controllers and the pressure inside the discharge tube is regulated with a back-pressure controller (25 mbar). The outflow of the gas from the discharge tube is directed to a MPC (effective path length of 31.2 m), which is regulated by another back-pressure controller to a pressure lower than in the discharge tube (16.5 mbar) to ensure a proper gas flow.

## Supplementary Information

Supplementary Figures.

## Data Availability

The data that support the findings of this study are available from the corresponding authors on reasonable request.
