# Supplementary material for: Mid-infrared supercontinuum-based Fourier transform spectroscopy for plasma analysis
Source: Sci Rep. 2022 Jun 10;12:9642. doi: 10.1038/s41598-022-13787-w (PMC9187747; doi:10.1038/s41598-022-13787-w)
Supplement: Supplementary file 1 — Supplementary Figures. [file 41598_2022_13787_MOESM1_ESM.pdf]

Mid-infrared supercontinuum-based Fourier transform spectroscopy for plasma analysis

R. Krebbers,[1] N. Liu,[1,2] K. E. Jahromi,[1] M. Nematollahi,[1] O. Bang,[3,4,5] G. Woyessa,[3] C. R. Petersen,[3,4] G. van Rooij,[6,7] F. J. M. Harren,[1] A. Khodabakhsh,[1,*] and S.M. Cristescu[1]

[1]Life Science Trace Detection Laboratory, Department of Analytical Chemistry and Chemometrics, Institute for Molecules and Materials, Radboud University, 6525 AJ Nijmegen, the Netherlands

[2]Laser Spectroscopy and Sensing Laboratory, School of Physics and Materials Science, Anhui University, 230601, Hefei, China

[3]DTU Fotonik, Department of Photonics Engineering, Technical University of Denmark, DK-2800 Kgs. Lyngby, Denmark

[4]NORBLIS ApS, Virumgade 35D, 2830 Virum, Denmark

[5]NKT Photonics A/S, Blokken 84, 3460 Birkerød, Denmark

[6]DIFFER - Dutch Institute for Fundamental Energy Research, De Zaale 20, 5612AJ Eindhoven, The Netherlands

[7]Faculty of Science and Engineering, Maastricht University, Paul Henri Spaaklaan 1, 6229 GS Maastricht, The Netherlands

*A.Khodabakhsh@science.ru.nl

Supplementary materials

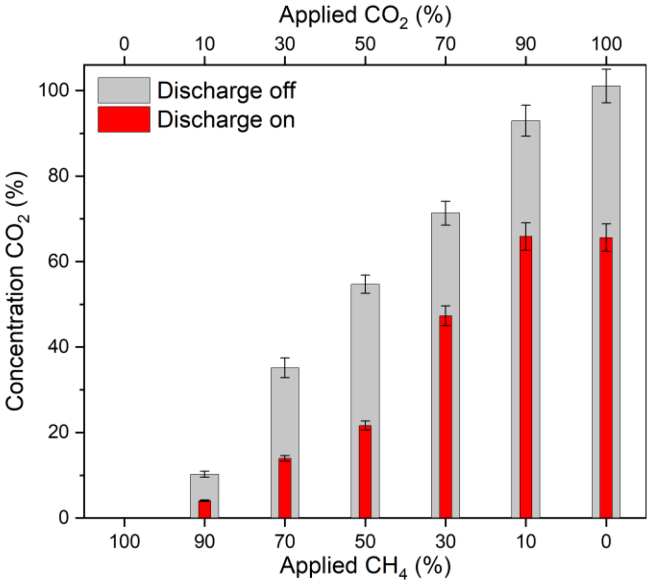

Supplementary Figure 1. Measured CO_2 concentrations with (in grey) and without (in red) the discharge applied for a varying CH_4/CO_2 ratio. The difference between the two indicates the amount of CO_2 that was consumed in the reaction within the discharge.

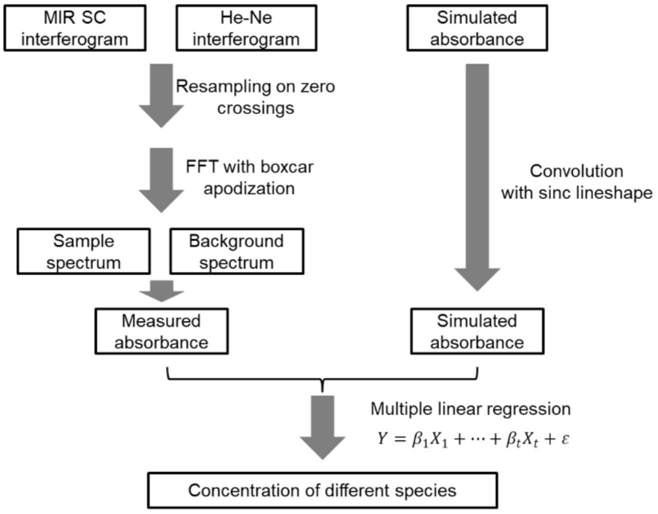

Supplementary Figure 2. Flowchart of the developed analysis algorithms.
